# End-Stage Renal Disease in a 29-Year-Old Male With Aneurysmal Arteriovenous Fistulas Status Post-Right-Kidney Transplant: A Case Report

**DOI:** 10.7759/cureus.41028

**Published:** 2023-06-27

**Authors:** Sharon Ekume, Sara Arfan, Muhammad Shahzad

**Affiliations:** 1 Department of Internal Medicine, Windsor University School of Medicine, Chicago, USA; 2 Department of Internal Medicine, AMITA Health Adventist Medical Center, Glen Oaks, USA

**Keywords:** public health education, screening guidelines, chronic kidney disease (ckd), end stage renal disease (esrd), hypertension

## Abstract

The occurrence of renal failure is higher among African Americans in comparison to individuals of other descents, indicating a disproportionate representation. Chronic kidney disease (CKD) poses a significant healthcare burden that disproportionately affects low-income and minority communities. There are various factors that drive the progression and deterioration of CKD to its advanced stages. These factors include genetic predispositions, socioeconomic status, barriers to medical care, and the patients’ own health beliefs and behaviors which impact their screening, risk factor control, and adherence to treatment. Earlier detection and management of hypertension can slow or halt the progression of CKD. This case report is on a case of a 29-year-old African American male with end-stage renal disease (ESRD) status-post right renal transplant. At 21 years old, the patient was diagnosed with benign essential hypertension which progressed from CKD to ESRD. Furthermore, at the age of 23 years old, he was requiring right renal transplants. We aim to shed light on the underlying predispositions that put this young patient at risk for CKD and related comorbidities. Lastly, to highlight dialysis-related complications from the treatment of ESRD and the impact of chronic illness on this patient’s overall health.

## Introduction

The National Kidney Foundation estimated that approximately 33% of African American adults are at risk of chronic kidney disease (CKD) and are disproportionately more likely to have kidney failure than their Hispanic or Caucasian counterparts [1]. CKD poses a significant healthcare burden from a public health point of view, disproportionately affecting low-income and minority populations. There are multiple factors that have become the focal point of the progression and worsening of CKD. Some of these factors include limitations to accessing high-quality medical care, patients’ health beliefs and behaviors, and social determinants of health [2]. Patients’ health beliefs and behaviors impact CKD screening, risk factor control, and adherence to therapy. Diabetes and hypertension are the two major risk factors for developing end-stage renal disease (ESRD) [3]. Studies have shown that earlier detection and treatment can limit or prevent the progression of CKD [4]. We report a case of a 29-year-old African American male with ESRD status-post right renal transplant, who had undiagnosed and uncontrolled hypertension. At 23, the patient’s hypertension progressed to ESRD. Our aim is to provide insight into genetic factors that predisposed this patient to CKD and related comorbidities. Lastly, dialysis-related complications from ESRD treatment and chronic illness's impact on mental health are discussed.

## Case presentation

**Figure 1:**
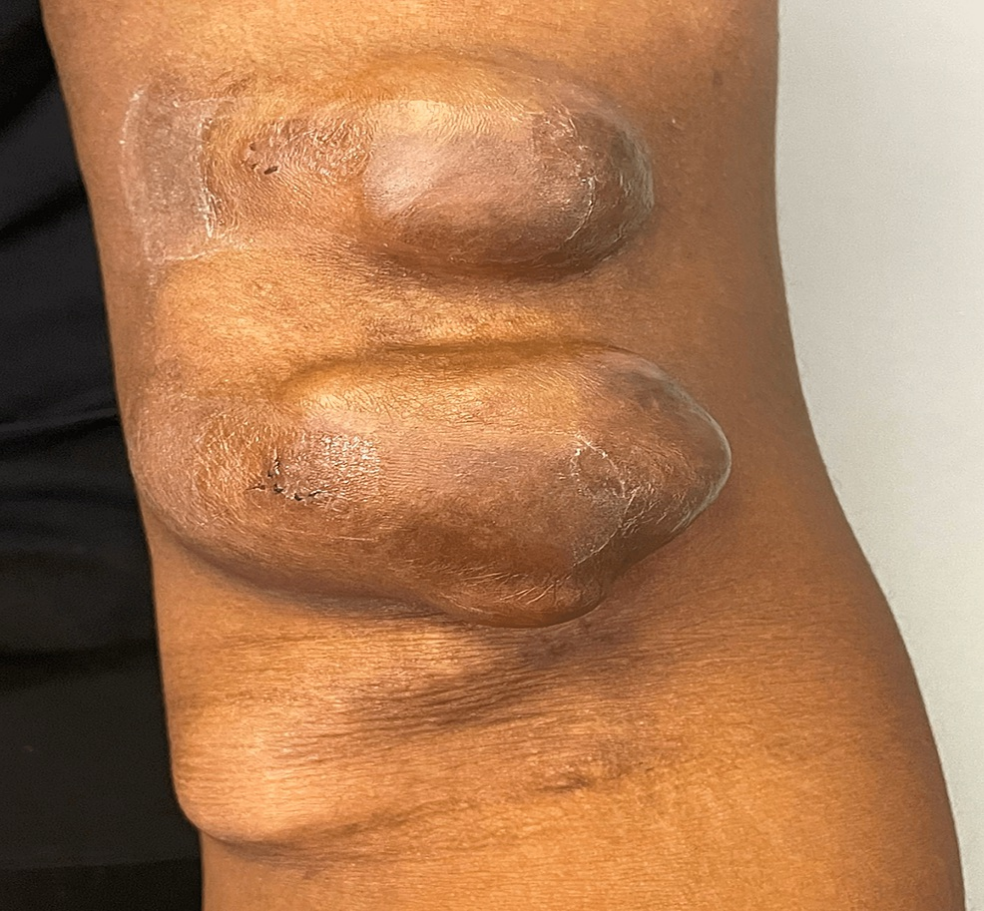
An anterior view of dialysis-related aneurysm formation from arteriovenous fistula-port (AVF), which ranges from 3cm, 5cm, and 7 cm with the last two being pulsatile on palpation. A total of 3 cm mass is a soft-non-tender mass, while the 5 cm and 7 cm masses were pulsatile and tender.

**Figure 2:**
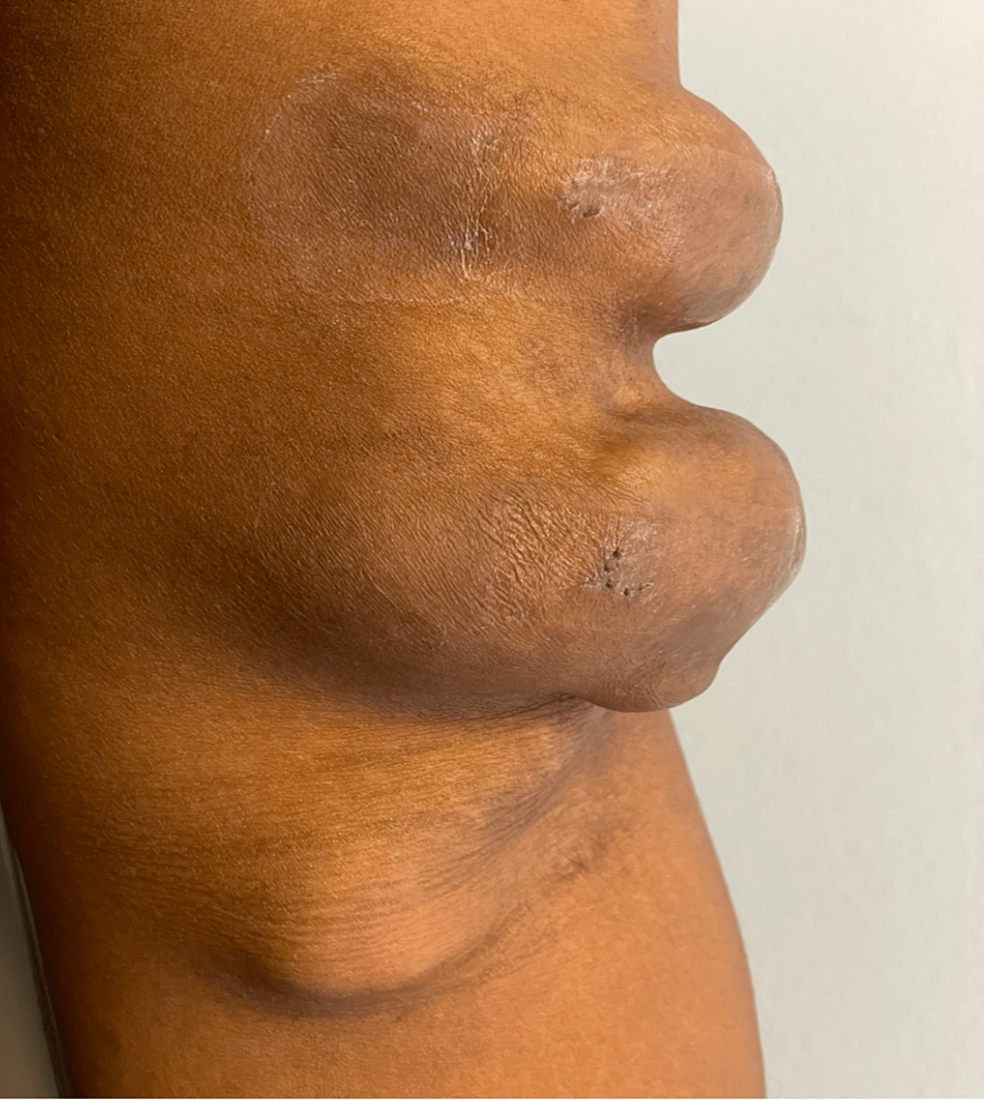
A mediolateral view of dialysis-related aneurysm formation from arteriovenous fistula-port (AVF), which ranges from 3 cm, 5 cm, and 7 cm in sizes with the last two pulsatile on palpation. A total of 3 cm mass is a soft-non-tender, while the 5 cm and 7 cm masses were pulsatile and tender.

We present the case of a 29-year-old African American male with a past medical history of ESRD status post right renal transplant, hyperparathyroidism status post partial resection, obesity, depression, hypertension, and hyperlipidemia. The patient presented to the outpatient clinic with three rapidly growing, painful masses ranging from 3 to 7 centimeters on his left upper extremity (Figures 1-2). He stated that the masses developed within the first year of starting dialysis and have since increased in size. Two of these masses were pulsatile in nature and tender to palpation and one was soft and non-tender on palpation. The pain progressively worsened over the past few months, was rated at a 9 out of 10 in severity, and was exacerbated by touch and movement. The overlying skin was shiny.

**Figure 3:**
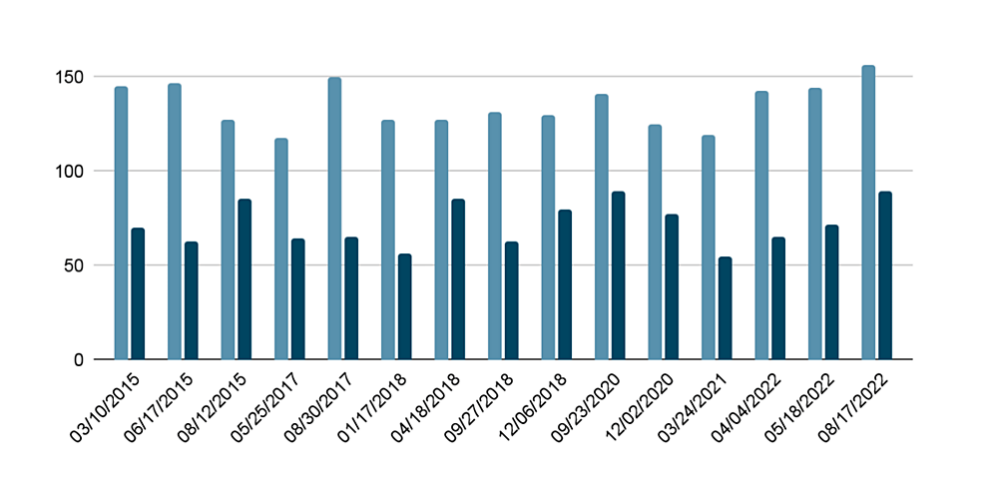
An eight-year timeline outlining the trends in the patient's systolic blood pressure (SBP) and diastolic blood pressure (DBP) from 2015 to 2022 while on medication for end-stage renal disease and hypertension.

The patient had a complex past medical history in that he was diagnosed with benign essential hypertension at the age of 21 years old with progression from CKD to ESRD by the age of 23 years old involving the right kidney. His family history was positive for hypertension and ESRD in his father, who passed away from unspecified complications of the disease as per the patient’s knowledge. The initial most encounter involved hospitalization due to suicide ideation and attempt. The patient denied tobacco use, alcohol consumption, and recreational or illicit drug use. He reported exercising three times weekly and following the DASH diet. Medications included calcium carbonate 500 mg PO QD, calcitriol 0.25 mcg PO TIW, aspirin 81 mg PO QD, tacrolimus 1 mg PO QD, prednisone 10 mg PO QD, metoprolol tartrate 50 mg PO BID, Nortriptyline HCL 25 mg PO QD, and gabapentin 300 mg PO BID. Hemodynamic parameters included a blood pressure of 156/89 mmHg, a pulse of 82 bpm, and a respiratory rate of 18 bpm. His body mass index (BMI) was 32.29 kg/m^2^. A review of systems (ROS) was non-contributory. During this encounter, his blood pressure ranged between 150/70 mmHg and 160/80 mmHg. Figure 3 details the trends in his blood pressure at several visits over the last eight years, from 2015 to 2022.

A complete blood count (CBC) and other laboratory testing were ordered to assess his renal function. Of note, were the elevated levels of serum blood urea nitrogen (BUN), 38.0 mg/dL, and creatinine (Cr) 4.32 mg/dL (Table 1). At the time of laboratory testing, the patient did not display any physical manifestations representative of renal pathology.

**Table 1:** Laboratory results on initial encounter in 2015 *Glomerular Filtration Rate (GFR) for non-African American individuals using the Modification of Diet in Renal Disease (MDRD) Study equation.

Test	Laboratory Value	Reference Range	Interpretation
White blood cell count	10,790 mL	5000-10,000 mL	Elevated
Red blood cell count	4.50 x 10^6 ^/µL	4.2-5.9 x 10^6 ^/µL	Normal
Hemoglobin	13.0 g/dL	13.8-17.2 g/dL	Decreased
Hematocrit	38.5%	41-50%	Decreased
Sodium	138 mmol/L	136-145 mmol/L	Normal
Potassium	2.8 mmol/L	3.5-5.0 mmol/L	Decreased
Glucose Level	104 mg/dL	70-100 mg/dL	Elevated
Blood urea nitrogen (BUN)	38 mg/dL	6-24 mg/dL	Elevated
Creatinine (Cr)	4.32 mg/dL	0.74-1.35 mg/dL	Elevated
Calcium	9.7 mg/dL	9-10.5 mg/dL	Normal
Glomerular filtration rate (GFR) (MDRD)*	*21.1 mL/min/1.73m^2^	>90 ml/min/1.73m^2^	Decreased
Creatinine Clearance	*25.2 mL/min	110-150 mL/min	Decreased
Intact Parathyroid hormone	344 pg/mL	10-65 pg/mL	Elevated
Protein Total, Serum	8.0 g/dL	6.0-7.8 g/dL	Elevated
Uric Acid	9.5 mg/dL	2.5-8 mg/dL	Elevated
Alkaline Phosphatase	97 units/L	36-92 units/L	Elevated
Lactate dehydrogenase	278 units/L	60-100 units/L	Elevated
Total Bilirubin	0.4 mg/dL	0.3-1.2 mg/dL	Normal

The patient is currently on the waitlist for a left renal transplant and was referred to his vascular access team for further assessment of his arteriovenous fistula (AVF). He was referred to a primary care clinic for follow-up and was started on dialysis four times weekly for the management of ESRD. At the young age of 26, this patient with an initial diagnosis of benign essential hypertension underwent a right renal transplant, was waitlisted for a left renal transplant, and received ongoing dialysis four times weekly.

## Discussion

Blacks and African Americans have the highest incidence of CKD compared with individuals from other racial backgrounds. This group makes up 31% of the CKD population in the United States [5]. The progression of CKD in African-Americans has been attributed to a combination of health and social risk factors such as diabetes mellitus, hypertension, and low socioeconomic status [6]. These statistics support the need for early prevention and detection of risk factors to prevent the progression of CKD. This case describes a 29-year-old African American male diagnosed with ESRD with aneurysmal AVF status post-right-kidney transplant and highlights the genetic predispositions and complications to improve medical literacy and raise awareness in this young age group. Additionally, we highlight the critical role of primary care physicians (PCPs) in the earlier detection, screening, and management to prevent devastating sequelae.

An investigation into the patient’s family history revealed that both his father and paternal grandfather suffered from hypertension and CKD and passed away in their early 50s due to complications of the disease. Individuals with first- and second-degree family members with a known history of ESRD are three times more likely to develop incident ESRD, have increased rates of renal dysfunction, undetected or uncontrolled hypertension, and are more likely to have higher BMI [7]. There is a dire need for public health screening protocols to take into account global risk factors such as age, race, genetic predisposition, socioeconomic status, and family history that may affect a high-risk patient like ours.

Currently, the diagnostic criteria for CKD require more than three months of declining kidney function as estimated by glomerular filtration rate (GFR) (normal: 125 mL/min/1.73 m^2^) and evidence of structural or functional kidney damage with pathological abnormalities [8]. In contrast, the 24-hour urine sample testing for albumin to creatinine ratio (ACR) (normal: <30 mg) was the initial diagnostic modality of choice [9]. Our patient had a high ACR of 19,440, GFR of 21.1 mL/min/1.73 m^2^, and a Cr of 4.32 mg/dL with a high total protein of 8.0 g/dL, all of which were indicative of renal deterioration prompting dialysis. Despite initial improvement, a steep decline in renal function was seen two years into dialysis treatment due to poor health choices and lifestyle, necessitating the need for a transplant. A 21-year-old patient with hypertension underwent a renal transplant at the age of 23, a devastating spiral of multiple comorbidities that ensued, which could have been avoided had there been better screening measures implemented for patients under his criteria. Furthermore, the patient developed three AVFs pulsatile aneurysms, measuring 6-8 cm on his left upper extremity, a dialysis-related infection. Dialysis which could have been avoided or delayed also predisposed this patient to further complications including aneurysms, pseudoaneurysms, bacteremia, and sepsis from frequent venipunctures and compromised vasculature [10]. Chang et al. performed a receiver operating characteristic (ROC) analysis which determined that patients who were on dialysis for longer than 5.7 years have a greater risk of developing vascular access aneurysms [11]. Our patient formed an aneurysm within the first year of hemodialysis. Chang et al. found that out of the 77,265 patients with advanced CKD who started permanent dialysis, 34,711 (55.2%) patients experienced at least one infection-related hospitalization [12].

CKD remains the leading cause of mortality in dialysis patients of all racial subgroups given the incidence of diabetes and hypertension in the dialysis population [5]. This effect is even more pronounced in African Americans, a group that has high infection-related mortality [13]. These complications make it crucial to monitor and educate at-risk patients to improve their medical literacy. Although effective methods of early detection tests and screening modalities are available, current guidelines do not account for young adults with strong genetic predispositions. Extensive testing and imaging are warranted in high-risk patients with various comorbidities and strong family histories. Low-income minority subgroups often seek treatment in the later stages of the disease due to a lack of adequate insurance coverage and compounded by the lack of thorough screening protocols, making it easier to overlook the diagnosis and prognosis in these groups. Health insurance coverage is of paramount importance, especially in ESRD patients who have high morbidity, mortality, and cost associated with their disease. This effect is pronounced in racial minorities with a low socioeconomic status, which directly contributes to subpar medical care, delayed diagnosis, and delayed management.

The first step to earlier recognition of CKD is taking a detailed history and physical, ordering appropriate lab work and imaging, and frequent follow-ups in a primary care setting. Patient involvement and medical literacy are key to compliance and in developing patient-physician rapport. ESRD is a growing burden on healthcare which can be minimized by earlier interventions that prevent its antecedent, CKD. High-risk patients should undergo systematic screening for timely intervention, which results in a decreased incidence of CKD complications and management (dialysis or transplant), thereby reducing the burden on healthcare resources and personnel. Notably, there is an urgent need for better healthcare policies and medical insurance coverage plans, which consider patient factors, including age, race, gender, and socioeconomic status.

## Conclusions

ESRD in young African American males is a life-altering diagnosis associated with several complications and an overall negative impact on the quality of life. The significance of reporting this case is to improve the screening and management of young at-risk individuals with a strong family history of the disease. The current guidelines do not account for young adults who do not present with structural or functional kidney damage. Earlier detection via screening modalities to prevent disease-related complications, improve quality of life, and decrease the cost and burden on healthcare resources and personnel can make a great impact.

## References

[1] Gaitonde DY, Cook DL, Rivera IM (2017). Chronic kidney disease: detection and evaluation. Am Fam Physician.

[2] Chen TK, Knicely DH, Grams ME (2019). Chronic kidney disease diagnosis and management: a review. JAMA.

[3] Jurkovitz CT, Li S, Norris KC, Saab G, Bomback AS, Whaley-Connell AT, McCullough PA (2013). Association between lack of health insurance and risk of death and ESRD: results from the Kidney Early Evaluation Program (KEEP). Am J Kidney Dis.

[4] Martins D, Agodoa L, Norris KC (2012). Hypertensive chronic kidney disease in African Americans: strategies for improving care. Cleve Clin J Med.

[5] McClellan WM, Satko SG, Gladstone E, Krisher JO, Narva AS, Freedman BI (2009). Individuals with a family history of ESRD are a high-risk population for CKD: implications for targeted surveillance and intervention activities. Am J Kidney Dis.

[6] Nicholas SB, Kalantar-Zadeh K, Norris KC (2015). Socioeconomic disparities in chronic kidney disease. Adv Chronic Kidney Dis.

[7] Levey AS, Coresh J, Balk E (2003). National Kidney Foundation practice guidelines for chronic kidney disease: evaluation, classification, and stratification. Ann Intern Med.

[8] Laster M, Shen JI, Norris KC (2018). Kidney disease among African Americans: a population perspective. Am J Kidney Dis.

[9] Xu D, Li J, Wang S, Tan Y, Liu Y, Zhao M (2022). The clinical and pathological relevance of waxy casts in urine sediment. Ren Fail.

[10] Jankovic A, Donfrid B, Adam J (2013). Arteriovenous fistula aneurysm in patients on regular hemodialysis: prevalence and risk factors. Nephron Clin Pract.

[11] Yu AJ, Norris KC, Cheung AK, Yan G (2017). Younger black patients have a higher risk of infection mortality that is mostly non-dialysis related: a national study of cause-specific mortality among U.S. maintenance dialysis patients. Hemodial Int.

[12] Chang CH, Fan PC, Kuo G (2020). Infection in advanced chronic kidney disease and subsequent adverse outcomes after dialysis initiation: a nationwide cohort study. Sci Rep.

[13] Price DA, Owen WF Jr (1997). African-Americans on maintenance dialysis: a review of racial differences in incidence, treatment, and survival. Adv Ren Replace Ther.

